# Mendelian randomization analysis reveals an independent causal relationship between four gut microbes and acne vulgaris

**DOI:** 10.3389/fmicb.2024.1326339

**Published:** 2024-02-02

**Authors:** Yujia Wu, Xiaoyun Wang, Wenjuan Wu, Jiankang Yang

**Affiliations:** ^1^School of Basic Medical Sciences, Dali University, Dali, China; ^2^Department of Dermatology, First Affiliated Hospital of Kunming Medical University, Kunming, China

**Keywords:** acne vulgaris, gut microbiota, Mendelian randomization analysis, short-chain fatty acid, inflammation

## Abstract

**Background:**

Numerous studies have suggested a correlation between gut microbiota and acne vulgaris; however, no specific causal link has been explored.

**Materials and methods:**

To investigate the possible causal relationship between gut microbiota and acne vulgaris, this study employed a large-scale genome-wide association study (GWAS) summary statistic. Initially, a two-sample Mendelian randomization (MR) analysis was utilized to identify the specific gut microflora responsible for acne vulgaris. We used the Inverse Variance Weighted (IVW) method as the main MR analysis method. Additionally, we assessed heterogeneity and horizontal pleiotropy, while also examining the potential influence of individual single-nucleotide polymorphisms (SNPs) on the analysis results. In order to eliminate gut microbiota with reverse causal associations, we conducted reverse MR analysis. Multivariate Mendelian randomization analysis (MVMR) was then employed to verify the independence of the causal associations. Finally, we performed SNP annotation on the instrumental variables of independent gut microbiota and acne vulgaris to determine the genes where these genetic variations are located. We also explored the biological functions of these genes through enrichment analysis.

**Result:**

The IVW method of forward MR identified nine gut microbes with a causal relationship with acne vulgaris (*p* < 0.05). The findings from the sensitivity analysis demonstrate the absence of heterogeneity or horizontal pleiotropy, and leave-one-out analysis indicates that the results are not driven by a single SNP. Additionally, the Reverse MR analysis excluded two reverse-correlated pathogenic gut microbes. And then, MVMR was used to analyze seven gut microbes, and it was found that Cyanobacterium and Family XIII were risk factors for acne vulgaris, while Ruminococcus1 and Ruminiclostridium5 were protective factors for acne vulgaris. After conducting biological annotation, we identified six genes (PLA2G4A, FADS2, TIMP17, ADAMTS9, ZC3H3, and CPSF4L) that may be associated with the pathogenic gut microbiota of acne vulgaris patients. The enrichment analysis results indicate that PLA2G4A/FADS2 is associated with fatty acid metabolism pathways.

**Conclusion:**

Our study found independent causal relationships between four gut microbes and acne vulgaris, and revealed a genetic association between acne vulgaris patients and gut microbiota. Consider preventing and treating acne vulgaris by interfering with the relative content of these four gut microbes.

## 1 Introduction

Acne vulgaris is a very common chronic inflammatory disease of the skin, manifested which is not only prone to occur during adolescence, but also persists in some adult patients (Kutlu et al., 2023). According to statistics, acne vulgaris has become the eighth most prevalent disease worldwide, affecting approximately 9% of the global population (Tan and Bhate, 2015). The pathogenesis of acne is not yet clear, but it is currently considered to be related to hyperactive sebaceous gland activity, aberrant keratinization of hair follicles and sebaceous glands, perturbations in the diversity of skin microbiota, inflammatory mechanisms, and organism immunity (Dréno, 2017; Dagnelie et al., 2022). Some acne vulgaris cases may be related to diet, hormones, genetics, cosmetics, medication, and emotions. The persistent inflammation caused by acne vulgaris can result in excessive pigmentation and the formation of scars after inflammation, which can have severe consequences for adults. This can affect the mental health of patients, leading to feelings of inferiority, depression, and even social isolation. Therefore, it is crucial for us to comprehend the potential factors that contribute to acne vulgaris and investigate its causes in order to generate fresh approaches for its treatment.

The microbiome of the digestive system, known as the gut microbiota, is a complex and constantly evolving community of microorganisms. It is related to the dynamics of human immune cells (Schluter et al., 2020). Dysregulation of the gut microbiota can lead to metabolic disorders of microorganisms in the intestines, which can in turn impact immune function. This indicates that gut microbiota can drive the immune system to exert regulatory effects. Recent studies have revealed that the impact of the gut microbiota extends beyond the gastrointestinal system, affecting the brain and skin as well. This has given rise to the concepts of the gut-brain axis and the gut-skin axis (Salem et al., 2018; Agirman et al., 2021). In the context of the gut-brain axis, research has found that regulating the gut and central nervous system (CNS), with a focus on their immune regulatory effects, can simultaneously affect inflammation in the gut, body, and brain (Ortega et al., 2023). Scientific research has identified a connection between imbalances in the gut microbiota and imbalances in the skin microbiota (Mahmud et al., 2022). Alterations in the gut microbiota have been linked to the development of inflammatory skin conditions (Polkowska-Pruszyńska et al., 2020). While some evidence suggests a correlation between the gut microbiota and acne vulgaris (Siddiqui et al., 2022), further investigation is needed to determine the exact nature of this relationship.

Mendelian randomization (MR) is a statistical method used to study the causal relationship between phenotype (exposure) and disease (outcome). It follows Mendel’s law of genetics, which states that allele genes are randomly distributed. MR analysis uses genetic variations related to the exposure as instrumental variables to infer the causal relationship with the outcome, thereby greatly reducing confounding and reverse causal associations (Sanderson et al., 2019). Multiple variable Mendelian randomization (MVMR) is the latest extension of MR, primarily using multiple potentially correlated exposures to assess the independent causal effects of each exposure on the outcome. MVMR is more robust when there are confounding factors between multiple exposures (Sanderson, 2021).

In this study, we used two samples MR analysis and MVMR analysis to investigate whether there is an independent causal relationship between different gut microbes and acne vulgaris. If it can be proven that a certain gut microbiota influences the occurrence and development of acne vulgaris, then intervention measures can be taken on the gut microbiota to effectively treat acne vulgaris.

## 2 Methods and materials

### 2.1 Study design

The main goal of this study is to investigate the causal relationship between gut microbiota and acne vulgaris. This study utilizes gut microbiota as an exposure factor and selects single nucleotide polymorphisms (SNPs) as instrumental variables (IVs) based on a threshold of *p* < 1 × 10^−5^. We conducted forward MR analysis using acne vulgaris as the outcome. Then, we perform reverse MR analysis using gut microbiota as the outcome. Finally, we used MVMR analysis to study the independent causal effects of different intestinal microbial clusters on acne vulgaris.

**Figure 1:**
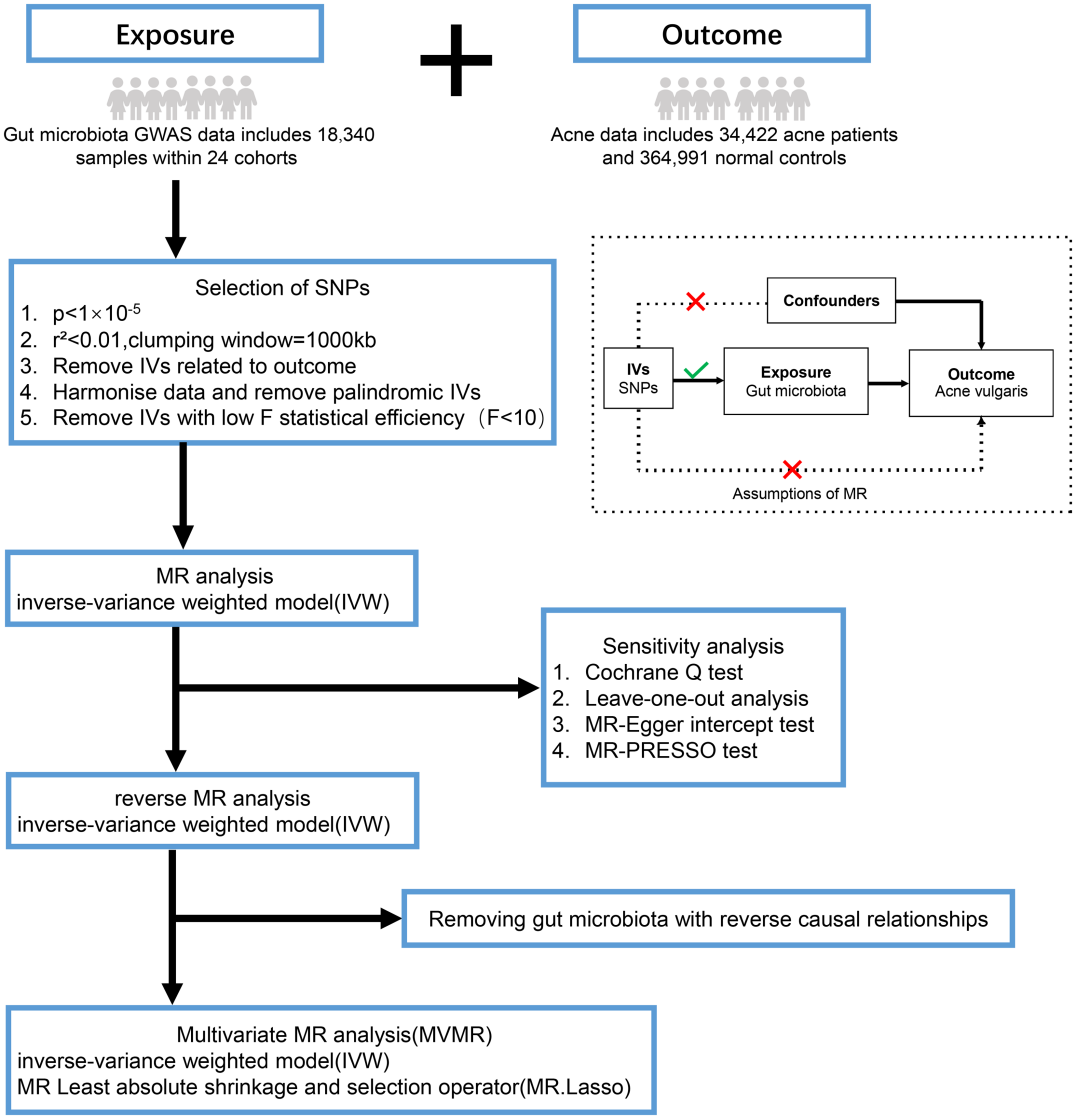
Overview of the analysis process of the causal relationship between the gut microbiota and acne vulgaris through MR analysis and MVMR analysis. GWAS, genome-wide association study; SNPs, single nucleotide polymorphisms; MR-PRESSO, mendelian randomization of pleiotropy residuals and outliers.

In this study, MR analysis must meet three main assumptions: (1) Relevance assumption: IVs are strongly associated with exposure and are independent of each other; (2) Exclusive assumption: IVs are irrelevant to the outcome; and (3) Independence assumption: IVs do not relate to the confusion factor (Birney, 2022) (Figure 1).

### 2.2 Data sources

Data for gut microbiota was obtained from a large-scale Genome-Wide Association Study (GWAS) (Kurilshikov et al., 2021). This study involved multiple cohorts, including 24 cohorts with a total of 18,340 individuals. Each cohort included only taxa that were present in more than 10% of the samples, resulting in a total of 211 taxa (131 genera, 35 families, 20 orders, 16 classes, 9 phyla). The included cohort adjusts the coherence variables for both gender and age in the calculation.

Acne vulgaris data are derived from GWAS studies and meta-analyses in European cohorts of EstBB, FinnGen and Lifelines, including 34,422 acne patients and 364,991 normal controls (Teder-Laving et al., 2023).

### 2.3 Selection of IVs

As the majority of gut microbiota do not have significant SNPs under the traditional threshold of *p* < 5 × 10^−8^, a relatively lenient statistical threshold (*p* < 1 × 10^−5^) is used in forward MR analysis to screen for SNPs related to the exposure (Feng et al., 2022; Liu et al., 2022). To meet MR’s assumption 1, linkage disequilibrium analysis was conducted using European reference data from the 1,000 Genomes project. Set the linkage disequilibrium related coefficient to *r*^2^ < 0.01, and the window size to 1,000 kb, to rule out genetic variations with linkage disequilibrium. Next, SNPs associated with exposure were extracted from the outcome. SNPs strongly correlated with the outcome (*p* < 1 × 10^−5^) were excluded and performed harmonization to eliminate SNPs with palindromic sequence and allele inconsistency (Zhang et al., 2021). Finally, we also computed the F statistics for each SNP to eliminate genetic variations with weak statistical power (*F* < 10) (Liu et al., 2022). The SNPs obtained above were used as IVs to study the effects of gut microbiota on acne vulgaris.

### 2.4 Forward MR analysis

We primarily utilized the Inverse Variance Weighted (IVW) method for conducting forward MR analysis. The IVW method is considered an ideal estimation method, as it can minimize the impact of confounding variables and provide unbiased estimates in the absence of horizontal pleiotropy (Bowden et al., 2017). Subsequently, we conducted a sensitivity analysis. We used Cochran’s Q test for heterogeneity analysis, where a *p*-value less than 0.05 is suggestive of possible heterogeneity in the IVs (Xiang et al., 2021). If heterogeneity was observed, the MR effect could be estimated through a direct random effects model. A leave-one-out sensitivity analysis was performed by sequentially removing each SNP and evaluating whether there were statistical differences in the result. If the results hardly change before and after removing single SNP, it suggests that single SNP may not have a significant impact on effect estimation. Additionally, the MR Egger method was implemented to test for horizontal pleiotropy. If an intercept term was identified in the MR-Egger intercept analysis with a *p* value for the intercept less than 0.05, then the study findings were deemed to have significant horizontal pleiotropy. The global test of Mendelian Randomization Pleiotropy RESidual Sum and Outlier (MR-PRESSO) is primarily utilized to evaluate horizontal pleiotropy and outliers.

### 2.5 Reverse MR analysis

To assess whether acne vulgaris has a causal impact on the gut microbiota, we employed a genome-wide significance threshold (*p* < 5 × 10^−8^) to screen for SNPs associated with acne vulgaris. These SNPs were used as IVs in reverse MR analysis, with acne vulgaris as the exposure and the gut microbiota as the outcome. The statistical methods used in reverse MR analysis were the same as those used in forward MR analysis.

### 2.6 MVMR analysis

Considering the possible interactions between gut microbiota, which may interfere with the results of univariate Mendelian randomization analysis, we proceeded with MVMR analysis. MVMR analysis aims to correct for the interactions between exposures by combining multiple exposures that may interact with each other (Sanderson, 2021).This analysis examines whether the influence of each significant gut microbiota on acne vulgaris, as identified in the univariate MR analysis, is independent.

In the bidirectional MR analysis, we identified several gut microbes that have an impact on acne vulgaris. These gut microbes were used as exposures to explore their relationship with the outcome. We selected IVs using a statistical threshold of *p* < 1 × 10^−5^ and removed SNPs in linkage disequilibrium (LD, r^2^ > 0.01, window size of 1,000 kb). We also excluded SNPs related to the outcome from the IVs. Additionally, we eliminated palindromic SNPs and SNPs with inconsistent alleles.

In MVMR analysis, we employ Least absolute shrinkage and selection operator (LASSO) regression to eliminate highly collinear exposures. Then, we utilize multivariable IVW as the primary method for conducting MVMR analysis, with MR LASSO being used to complement the IVW results. To enhance the robustness of causal relationships, we also perform sensitivity analysis using the MR-PRESSO method.

### 2.7 Annotation of biology

We selected IVs for significant gut microbiota from MVMR and subsequently linked these SNPs to specific genes. IVs of acne vulgaris disorders were also mapped onto genes. We then used version 12.0 of STRING to identify protein–protein interaction (PPI) networks of mapped genes that had independent causal relationships between gut microbiota and acne vulgaris, and identified core genes. Finally, we performed GO enrichment analysis on these core genes.

### 2.8 Data analysis

All statistical analyses were performed in R, version 4.3.1. Univariate Mendelian randomization analyses were primarily conducted using the “TwoSampleMR” software package (version 0.5.7), while multivariate Mendelian randomization analyses were primarily carried out with the “MendelianRandomization” software package. MR-PRESSO tests were performed using the “MRPRESSO” package. GO enrichment analysis was performed using software packages such as “clusterProfiler” and “org.Hs.eg.db.”

## 3 Result

### 3.1 Selection of IVs

In this study, the exposed data came from 211 gut microbes, including five biological classifications: phyla, class, order, family, and genus. The outcome data were collected from 34,422 acne patients and 364,991 normal people. In the exposed data processing, 2,129 SNPs were selected as IVs after removing the linkage disequilibrium and deleting the SNPs associated with the outcome according to the threshold of *p* < 1 × 10^−5^. We collected information about SNPs, including effector alleles, the effect of SNP on phenotype (β), effect allele frequency (EAF), standard error of β value (SE), and *p*-values. In addition, we also calculated the *F*-value for each SNP, and the F-statistic value for the SNPs used as IVs was greater than 10, further indicating the robustness of the IVs we used.

### 3.2 Forward MR analysis

After setting significant difference criteria (*p* < 0.05) based on the IVW method, we found that nine gut microbes have a causal effect on acne vulgaris, including one phylum, two families, and six genera (Supplementary Table S1).

**Figure 2:**
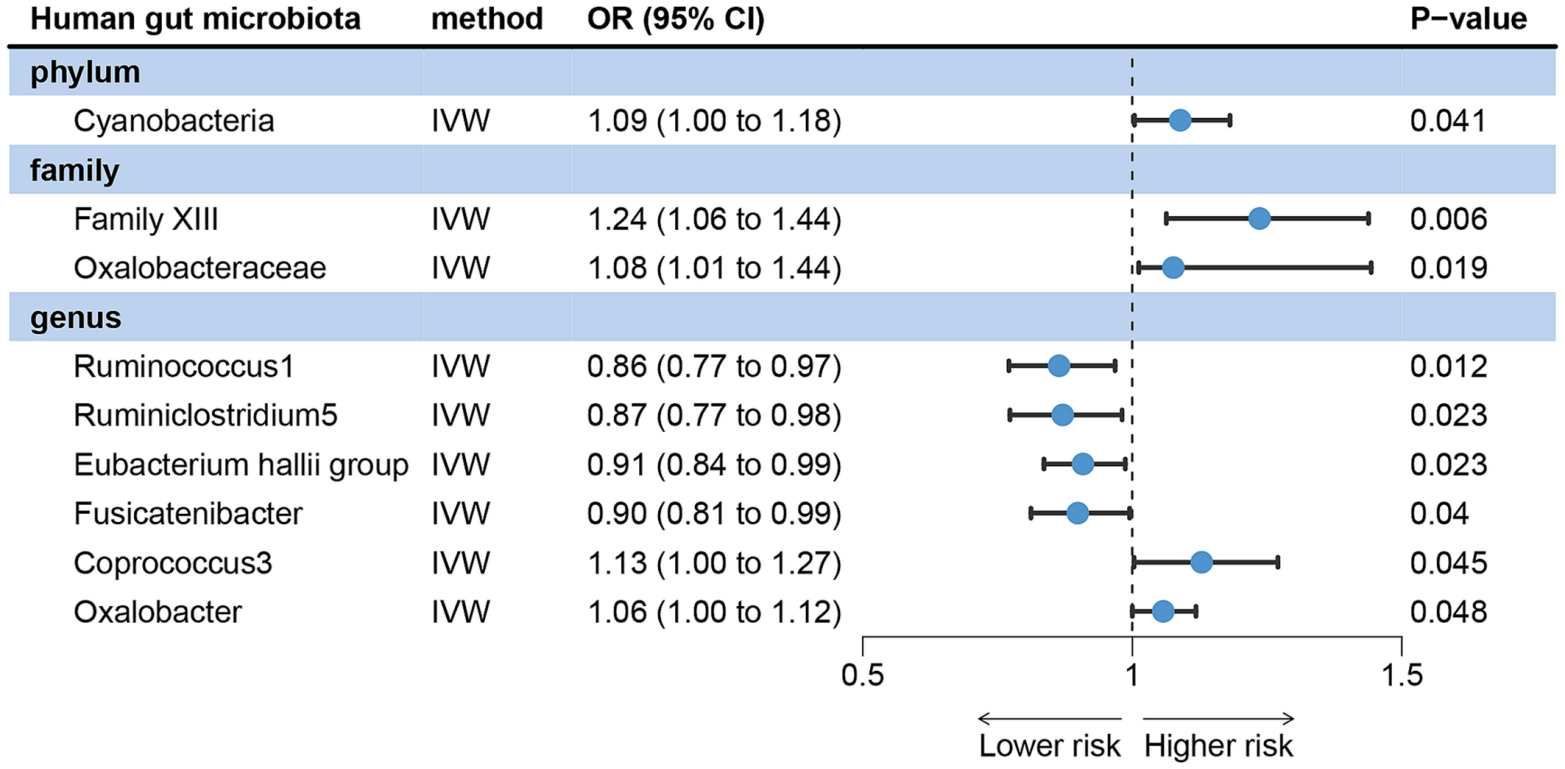
Forward MR analysis revealed potential causal relationships between gut microbiota with acne vulgaris risk. IVW, inverse variance weighted; OR, odds ratio; CI, confidence interval.

IVW analysis showed that Family XIII [odds ratio (OR) = 1.24, 95% confidence interval (CI): 1.06 ~ 1.44, *p* = 0.006], Oxalobacteraceae (OR = 1.08, 95%CI: 1.01 ~ 1.14, *p* = 0.012), Cyanobacteria (OR = 1.09, 95%CI: 1.00 ~ 1.18, *p* = 0.041), Coprococcus3 (OR = 1.13, 95%CI: 1.00 ~ 1.27, *p* = 0.045) and Oxalobacter (OR = 1.06, 95%CI: 1.00 ~ 1.12, *p* = 0.048) could increase the risk of acne vulgaris. Ruminococcus1 (OR = 0.86, 95%CI: 0.77 ~ 0.97, *p* = 0.012), Ruminiclostridium5 (OR = 0.87, 95%CI: 0.77 ~ 0.98, *p* = 0.023), *Eubacterium hallii* group (OR = 0.91, 95%CI: 0.84 ~ 0.99, *p* = 0.023) and Fusicatenibacter (OR = 0.88, 95%CI: 0.81 ~ 0.99, *p* = 0.040) were the protective factors of acne vulgaris (Figure 2).

The Cochran’s Q test for IVW showed no heterogeneity among IVs (Table 1). Leave-one-out analysis did not detect any abnormal SNPs (Supplementary Figure S1). The MR-Egger regression intercept (*p* > 0.05) and the results of the MR-PRESSO global test (*p* > 0.05) both indicate the absence of horizontal pleiotropy (Table 1).

**Table 1:** Heterogeneity and pleiotropy for forward Mendelian analysis.

Exposure: human gut microbiota	NSNPs	Outcome	Heterogeneity test			Pleiotropy test	
			Method	Cochran’s Q	*p*-value	Egger intercept (*p*-value)	MR PRESSO global test (*p*-value)
Phylum							
Cyanobacteria	8	Acne	IVW	4.734	0.692	0.951	0.664
Family							
Family XIII	6	Acne	IVW	4.512	0.478	0.402	0.524
Oxalobacteraceae	14	Acne	IVW	17.883	0.162	0.906	0.189
Genus							
Ruminococcus1	8	Acne	IVW	3.188	0.867	0.619	0.890
Ruminiclostridium5	10	Acne	IVW	7.036	0.633	0.984	
Eubacterium hallii group	13	Acne	IVW	9.692	0.643	0.272	0.661
Fusicatenibacter	19	Acne	IVW	25.108	0.122	0.142	0.122
Coprococcus3	9	Acne	IVW	4.466	0.813	0.434	0.828
Oxalobacter	11	Acne	IVW	7.197	0.707	0.617	0.717

IVW, inverse variance weighted; MR PRESSO, MR pleiotropy residual sum and outlier.

### 3.3 Reverse MR analysis

In the reverse MR Study, 25 SNPs were selected as IVs for acne vulgaris, and all had F-statistics greater than 10.

**Figure 3:**
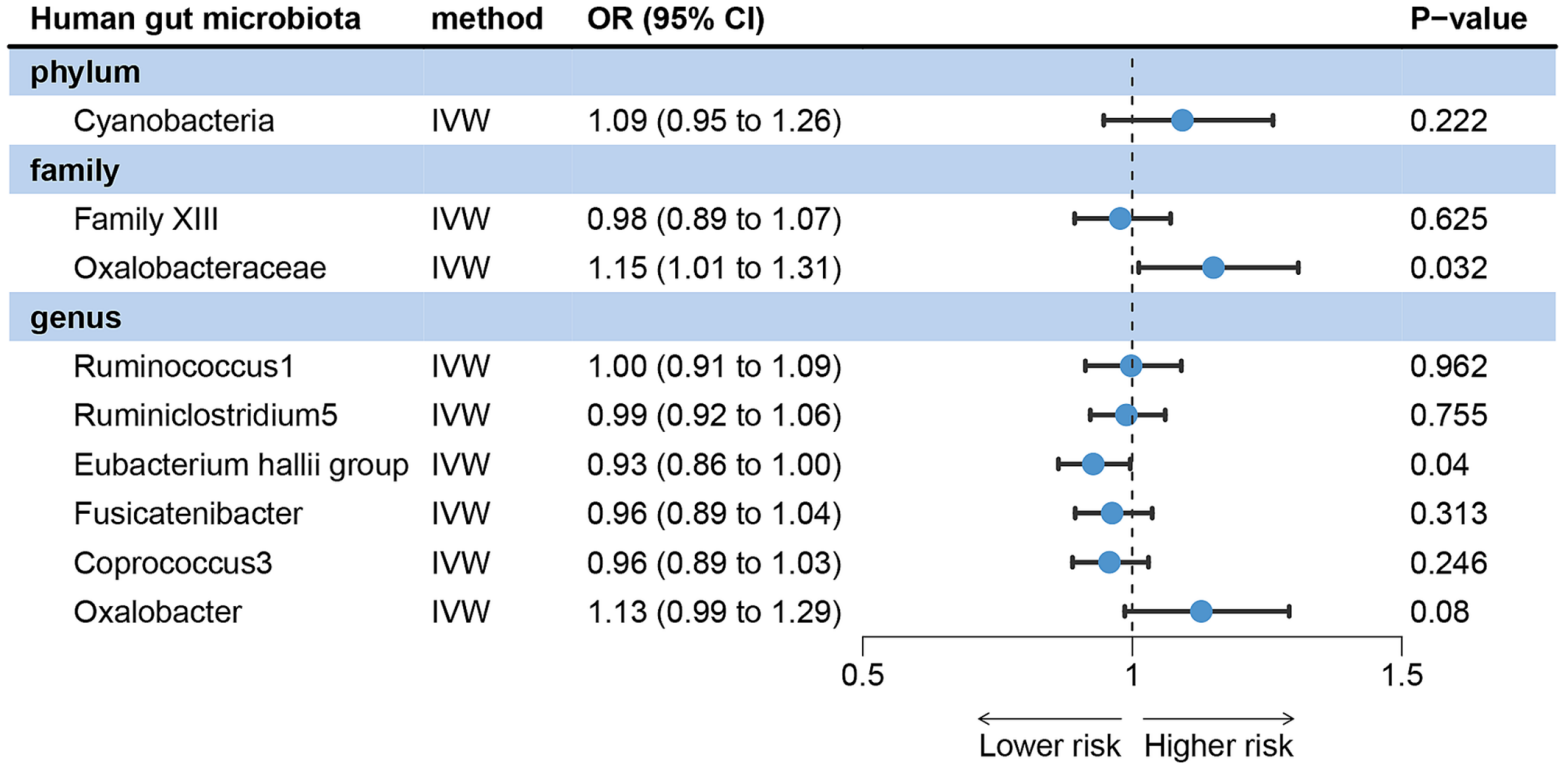
Reverse MR analysis revealed potential causal relationships between acne vulgaris and the gut microbiota.

The IVW analysis results revealed that acne vulgaris did not have a causal impact on Cyanobacteria, Family XIII, Ruminococcus1, Ruminiclostridium5, Fusicatenibacter, Coprococcus3, and Oxalobacter in the gut microbiota. However, it did have a causal effect on Oxalobacteraceae and the *Eubacterium hallii* group (Figure 3). The Cochran’s Q test, MR-Egger, and MR-PRESSO global tests showed no significant heterogeneity and horizontal pleiotropy (Supplementary Table S2).

### 3.4 MVMR analysis

Based on the findings acquired from the bidirectional analysis of MR, it has been determined that seven gut microbes have a causal relationship with acne vulgaris. Following a series of quality control and employing a relatively lenient criterion (*p* < 1 × 10^−5^), a total of 67 SNPs were chosen from these seven gut microbes to serve as IVs for the multivariate MR analysis.

**Figure 4:**
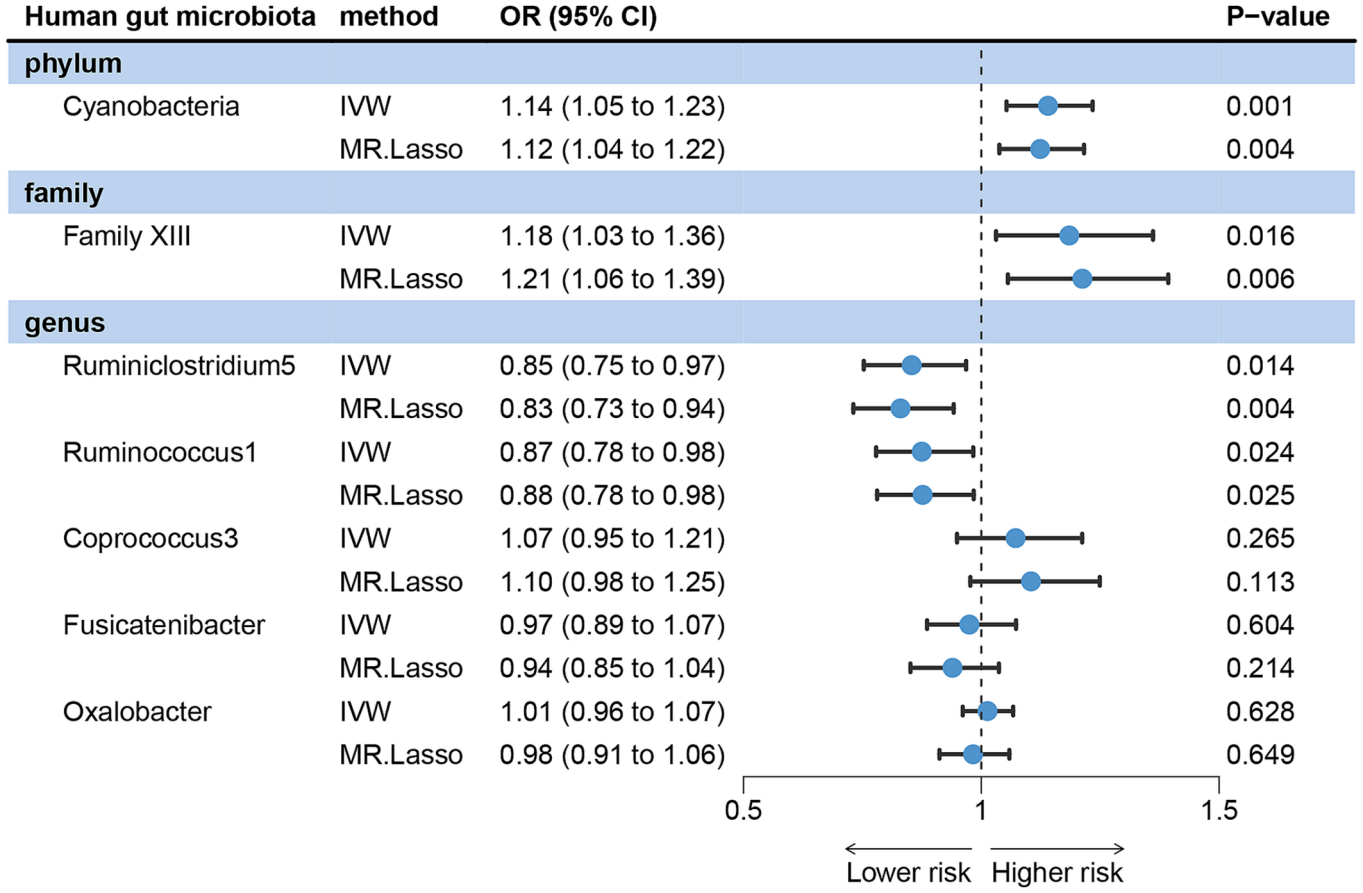
Forest plots showing independent causal associations between gut microbiota and acne vulgaris.

IVW analysis method showed that Cyanobacteria, Family XIII, Ruminiclostridium5 and Ruminococcus1 could have a direct causal effect on acne independently of other gut microbes. After adjusting for these four gut microbes, we observed that the direction of the causal effect was consistent with that of the forward MR. The positive association was discovered between Cyanobacteria (OR = 1.14, 95% CI: 1.05 ~ 1.23, *p* = 0.001) and Family XIII (OR = 1.18, 95% CI: 1.03 ~ 1.36, *p* = 0.016) with the risk of acne vulgaris. On the other hand, Ruminiclostridium5 (OR = 0.85, 95% CI: 0.72 ~ 0.97, *p* = 0.014) and Ruminococcus1 (OR = 0.87, 95% CI: 0.78 ~ 0.98, *p* = 0.024) were negatively associated with the risk of acne vulgaris (Figure 4).

In MVMR analysis, the effects of Oxalobacter, Fusicatenibacter, and Coprococcus3 on acne vulgaris became less significant, indicating that these three gut microbes may be influenced by other microbiota rather than independent influencing factors. MR-LASSO regression analysis gave similar and significant estimates (Figure 4). Sensitivity analysis showed no pleiotropy and outliers (Supplementary material S1).

### 3.5 Annotation of biology

**Figure 5:**
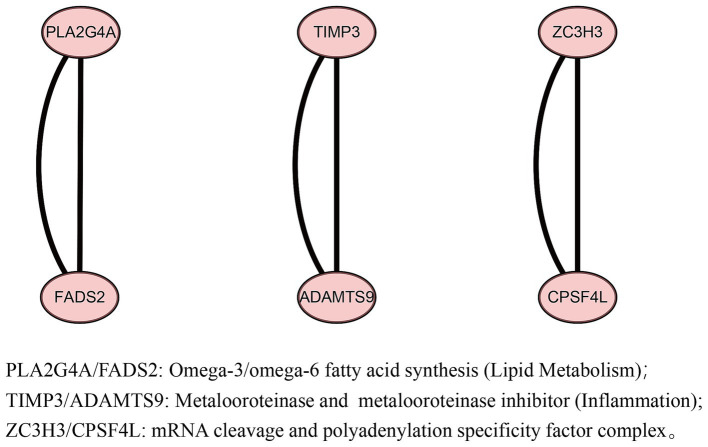
Protein–protein interaction (PPI) network between four gut microbes and acne vulgaris.

Four gut microbes with independent causal relationships were identified through MVMR. We found that these IVs from four gut microbes could mapped to 16 genes after a series of quality controls (Supplementary material S2). Mapping the IVs from acne vulgaris to 13 genes (Supplementary material S2). After conducting a PPI network analysis using the STRING, these 29 genes were found to form three interconnected networks (Figure 5), and identified six core genes (PLA2G4A, FADS2, TIMP3, ADAMTS9, ZC3H3, and CPSF4L). GO enrichment analysis and function annotation showed that PLA2G4A/FADS2 was enriched in fatty acid metabolism, TIMP3/ADAMTS9 was associated with inflammation, and ZC3H3/CPSF4L was enriched in mRNA processing pathway.

## 4 Discussion

For this study, we gathered extensive GWAS data pertaining to acne vulgaris and gut microbiota. Utilizing bidirectional MR analysis and MVMR analysis, we sought to investigate the causal impacts of 211 gut microbes on acne vulgaris. Notably, this is also the first time that MR analysis has been used to study the relationship between acne vulgaris and gut microbiota. Forward MR results showed that Family XIII, Oxalobacteraceae, Cyanobacteria, Coprococcus3 and Oxalobacter were risk factors for acne vulgaris. Ruminococcus1, Ruminiclostridium5, *Eubacterium hallii* group and Fusicatenibacter were the protective factors for acne vulgaris. However, after reverse MR Analysis, it was found that acne vulgaris had a causal effect on Oxalobacteraceae and *Eubacterium hallii* group. Therefore, we considered that Oxalobacteraceae and *Eubacterium hallii* group might interact with acne vulgaris. The comprehensive two-sample Mendelian analysis results indicated that seven gut microbes have a causal effect on acne vulgaris. To avoid the interference of the presence of confounding factors, we then performed MVMR analysis of seven gut microbes. According to the results of MVMR analysis, Cyanobacteria, Family XIII, Ruminiclostridium5, and Ruminococcus1 have the ability to directly influence acne vulgaris, independent of other gut microbes.

The gut microbial flora colonizes the human gut at birth, and with time and age, the various microorganisms interact to balance and eventually become in a symbiotic state, which plays an immune role. If the diversity of gut microbiota changes, it can lead to the disruption of mucosal immune tolerance, which in turn affects skin health. When stimulated, a type of innate lymphoid cells (ILCs) located in epithelial cells can be activated to produce cytokines, thereby playing a defensive or pathogenic role in inflammation. Epithelial cells also express various pattern recognition receptors (PRRs), including Toll like receptors (TLRs). Under anti-inflammatory stimulation, it also produces chemokines for bone marrow cells and lymphocytes, thereby mobilizing the immune system (Shi et al., 2017). Acne vulgaris is a disease characterized by skin inflammation. Research has found that *Propionibacterium acnes* induces acne inflammation mainly through a member of TLRs-TLR2. Recently, it has been discovered that there is a complex interaction between the gut and skin, namely the gut dermis axis, which plays a crucial role in the inflammatory immune response (Salem et al., 2018). Studies have shown that skin inflammation may be caused by small changes in a certain bacterial species in the gut microbiome (Ainonen et al., 2022), and experiments have shown that the gut microbiome affects the development of acne through the mTOR pathway (Noureldein and Eid, 2018). According to numerous studies in recent years, the mTOR cascade can respond to many stimuli to regulate signaling pathways and important cellular biology functions, such as growth, survival, proliferation, and cell senescence, which are dysregulated in a variety of diseases (Jia et al., 2023; Xia et al., 2023). In addition, some studies have shown that acne vulgaris can increase endotoxemia and intestinal permeability, thereby altering the intestinal barrier and causing the imbalance of intestinal microbiota (Thompson et al., 2020). This was also demonstrated in a study in which acne vulgaris patients had distinct gut microbiome components (Deng et al., 2018). So, we considered that there might be a reciprocal relationship between acne vulgaris and gut microbiota. This hypothesis can be used to explain the significance of Oxalobacteraceae and *Eubacterium hallii* group in bidirectional MR Analysis, indicating that Oxalobacteraceae and *Eubacterium hallii* group interact with acne vulgaris.

There may be interactions between gut microbiota, and we hope to find microbiota that act independently on the occurrence and development of acne vulgaris. Through the analysis of MVMR, we have discovered that four gut microbes have independent causal effects on acne vulgaris. Cyanobacteria and Family XIII are risk factors for acne vulgaris, while Ruminiclostridium5 and Ruminococcus1 are protective factors for acne vulgaris.

Cyanobacteria is a large group in the gut microbiome and includes oxyphotosynthetic cyanobacteria and non-photosynthetic cyanobacteria-like black algae, which were recently identified in the human gut (Soo et al., 2014). Lipopolysaccharide (LPS) from the outer membrane of cyanobacteria has a unique structure and can trigger TLR4 to induce immune response (Hu and Rzymski, 2022). TLR4, a member of the TLR family, plays a crucial role in recognizing the pathogen-associated molecular pattern (PAMP) and damp-associated molecular pattern (DAMP) of microorganisms and native molecules. The activation of TLR4 in the MyD88 pathway involves the binding of TIRAP to the TIR domain of TLR4, leading to the activation of the IκB kinase complex. Consequently, the transcription of inflammatory genes is initiated, thereby contributing to the development of inflammation (Burns and Yusuf, 2014). It is noteworthy that the excessive activation of TLR4 has been associated with autoimmune and inflammatory diseases (Kondo et al., 2012). Additionally, the LPS derived from cyanobacteria has been implicated in various human diseases such as skin diseases, gastrointestinal diseases, respiratory diseases, and allergic diseases (Durai et al., 2015). Studies have identified a correlation between increased abundance of cyanobacteria in the gut and several diseases, including precancerous lesions of colorectal cancer, human norovirus infection, irritable bowel syndrome, allergic rhinitis, and lung cancer (Sarangi et al., 2017; Zhang et al., 2018; Zhu et al., 2020; Chumpitazi et al., 2021; Xiong et al., 2021). Similar to the results in the current study, we found for the first time that Cyanobacteria were associated with the development of acne vulgaris. Cyanobacteria is a risk factor for acne vulgaris, indicating that Cyanobacteria may be involved in regulating the progression of acne vulgaris through the stimulation of LPS.

We found that there are few studies on Family XIII, and the definition of Family XIII is not very detailed. In a study, it was observed that there is an increase in the abundance of Family XIII in patients with multiple sclerosis (Galluzzo et al., 2021). Multiple sclerosis is a chronic demyelinating inflammatory disease of the central nervous system. Therefore, we considered that Family XIII of gut microbiota might be involved in some inflammatory reactions in human body. This is the first time we have discovered the correlation between Family XIII and acne vulgaris. The specific mechanism is yet to be explored.

Certain research has discovered that there is a diminished abundance of Ruminococcus in inflammatory conditions like psoriasis, allergic diseases, and inflammatory bowel diseases. A study showed a significant reduction in the family Ruminococcaceae in patients with acne vulgaris and suggested that disruption of gut microbes may contribute to early inflammation in acne vulgaris (Deng et al., 2018). Ruminococcus belongs to the Ruminococcaceae (Molinero et al., 2021). This is consistent with our conclusion that the pathogenesis of acne vulgaris may be caused by the reduction of Ruminococcus1 in gut microbiota. The research on the gut microbiota and sex hormones has revealed that the gut microbiota can influence the levels of sex hormones, thereby affecting the immune development of the host (Korpela et al., 2021). Testosterone is an important male hormone in the human body, and Ruminococcus has a negative correlation with the increase of testosterone levels in healthy women (d'Afflitto et al., 2022). Androgens can induce excessive secretion of lipid in sebaceous glands and have proinflammatory effects on cortical cells. Current studies have suggested that acne vulgaris is an androgen-dependent inflammatory disease of sebaceous glands. *In vitro* experiments have also proved that androgens can promote the formation of acne vulgaris and promote the biosynthesis of growth factors in dermal fibroblasts (Hu et al., 2021). In the treatment of acne vulgaris, drugs that inhibit androgen receptor signaling are also currently selected (Rao et al., 2021). This led us to hypothesize that the reduction of Ruminococcus1 may increase the level of testosterone in the body, and then lead to the occurrence of acne vulgaris.

Ruminiclostridium5 is a beneficial bacterium that belongs to the phylum Firmicutes and it was first reported to be associated with acne vulgaris. It has been observed that this bacterium plays a role in the production of short-chain fatty acids (SCFAs). Research has indicated that SCFAs have a protective effect against inflammatory diseases such as colitis, arthritis, and allergies (Kim et al., 2014), playing an important role in maintaining immune homeostasis (Wang et al., 2023). The bacterium is capable of degrading polysaccharides through self-secretion, resulting in the production of multi-enzyme complexes. These complexes then generate short-chain fatty acids like butyrate and acetate, which contribute to the anti-inflammatory effects of Ruminiclostridium5 (Yuan et al., 2022). Especially butyrate salts, it can maintain the integrity of mucous membranes, prevent the symbiotic expansion of potential pathogenic bacteria in the intestines, inhibit the expression of destructive cytokines, and regulate immunity and inflammation (Xiao et al., 2020). This could be the mechanism by which Ruminiclostridium5 reduces the risk of acne vulgaris. Taken together with our MR Findings suggesting a causal relationship between Ruminiclostridium5 and acne vulgaris is plausible, we can achieve acne vulgaris prevention and control by increasing the abundance of Ruminiclostridium5 in various ways.

The biological annotation analysis indicates that we have identified 3 pairs of associated genes between gut microbiota and acne vulgaris, including PLA2G4A/FADS2, TIMP3/ADAMTS9 and ZC3H3/CPSF4L. PLA2G4A is a member of the cytosolic phospholipase A2 family, which generates arachidonic acid derivatives and is upregulated in atopic and allergic environments (Mouchlis and Dennis, 2019). Numerous research studies have provided evidence that infection caused by *Staphylococcus aureus* enhances the activity of PLA2G4A (Hardman et al., 2017). FADS2 is a gene associated with lipid metabolism, upregulation of its expression can induce pro-inflammatory sebaceous gland activity (Zouboulis and Angres, 2021). Recent genome-wide association studies and meta-analysis have shown that FADS2 is a risk gene for acne vulgaris (Teder-Laving et al., 2023). TIMP-3 is a secreted protein that has a broad inhibitory effect on matrix metalloproteinases (MMPs). Research has shown that TIMP-3 inhibits the expression of inflammatory cytokines upregulated by ultraviolet radiation in human keratinocytes (Park et al., 2018). ADAMTS9 is a member of the protein family that encodes ADAMTS (a disintegrin and metalloproteinase with a platelet reactive protein motif). In a study on polycystic ovary syndrome, it was found that the level of IL-17A in patients was negatively correlated with ADAMTS9 (Karakose et al., 2016). At present, there are few studies on the ZC3H3 and CPSF4L genes. Some studies have found that ZC3H3 is involved in mediating nuclear RNA decay (Silla et al., 2020), and CPSF4L may be associated with obesity (Dhana et al., 2018). Currently, research indicates that the key mechanisms involved in the development of acne vulgaris include increased sebum production and changes in the composition of sebum fatty acids (Moradi Tuchayi et al., 2015). These findings align with the outcomes derived from GO enrichment analysis. In particular, the fatty acid metabolism pathway shows enrichment of PLA2G4A/FADS2, which further supports the correlation between four gut microbes and acne vulgaris.

The primary advantage of this investigation resides in the fact that the utilization of the MR Approach lessened confounding variables and the obstruction of reverse causality on the outcomes, which may be more persuasive than customary observational investigations. However, several limitations should be acknowledged. First of all, the data in this study were from people of European ancestry, and the situation of other ethnic groups is not clear. Next, the absence of sex and age statistics hindered the possibility of conducting further subgroup analyses. Third, we did not consider multiple testing given that multiple testing correction may be too conservative and may omit potential gut microbiota causally related to acne vulgaris. Fourthly, due to data limitations, our study cannot confirm whether the gut microbiota, which is causally associated with acne, is affected by antibiotic.

## 5 Conclusion

This study has discovered four gut microbes that have a causal relationship with acne vulgaris, three of which (Cyanobacteria, Family XIII, Ruminiclostridium5) have not been reported in previous studies, which allows us to further investigate the affected mechanisms of these three gut microbes in the future. We also identified six genes associated with the gut microbiota and acne vulgaris, revealing a genetic association between acne patients and the gut microbiota. At present, the use of probiotics to change the intestinal flora in the treatment of acne vulgaris is becoming more and more popular, which also makes us wonder whether we can change the relative abundance of these four gut microbes in the human body to play a role in the treatment or the intervention of acne vulgaris.

## Data availability statement

The original contributions presented in the study are included in the article/Supplementary material, further inquiries can be directed to the corresponding authors.

## Author contributions

YW: Writing – original draft, Conceptualization, Data curation, Formal analysis, Methodology, Resources, Visualization, Writing – review & editing. XW: Writing – original draft, Data curation, Formal analysis, Investigation, Methodology, Validation, Visualization. WW: Writing – review & editing, Conceptualization, Funding acquisition, Project administration, Resources, Supervision, Validation. JY: Writing – review & editing, Conceptualization, Data curation, Formal analysis, Funding acquisition, Investigation, Methodology, Project administration.
