# Supplementary material for: Mendelian randomization analysis reveals an independent causal relationship between four gut microbes and acne vulgaris
Source: Front Microbiol. 2024 Feb 2;15:1326339. doi: 10.3389/fmicb.2024.1326339 (PMC10869500; doi:10.3389/fmicb.2024.1326339)
Supplement: Supplementary file 1 [file Data_Sheet_1.docx]

| Sensitivity analysis | | | | | | |
| --- | --- | --- | --- | --- | --- | --- |
| Exposure | MR Analysis | Causal | Estimate | Sd | T-stat | P-value |
| genus Oxalobacter | Outlier-corrected | NA | NA | NA | NA | NA |
| genus Ruminococcus1 | Outlier-corrected | NA | NA | NA | NA | NA |
| family Family XIII | Outlier-corrected | NA | NA | NA | NA | NA |
| genus Fusicatenibacter | Outlier-corrected | NA | NA | NA | NA | NA |
| genus Ruminiclostridium5 | Outlier-corrected | NA | NA | NA | NA | NA |
| phylum Cyanobacteria | Outlier-corrected | NA | NA | NA | NA | NA |
| genus Coprococcus3 | Outlier-corrected | NA | NA | NA | NA | NA |

MR-PRESSO results_Global Test

MR-PRESSO results_Global Test_RSSobs:76.58783

MR-PRESSO results_Global Test_Pvalue: 0.44

Cochran’s Q test_Qstat:52.67323

Cochran’s Q test_Pvalue:0.7063202

| The genes involved in mapping the instrumental variables of gut microbiota | |
| --- | --- |
| SNP | gene |
| rs10167839 | UBE2E3 |
| rs11783695 | ZC3H3 |
| rs17781867 | CPSF4L |
| rs6105066 | TASP1 |
| rs6493760 | none |
| rs7117576 | ZBTB16 |
| rs78572139 | none |
| rs78613526 | none |
| rs10501370 | none |
| rs10798999 | CSMD2 |
| rs10808115 | none |
| rs117748144 | none |
| rs13116360 | none |
| rs17474256 | none |
| rs28584818 | ADAMTS9 |
| rs60254196 | ZNF398 |
| rs630939 | none |
| rs6550770 | none |
| rs74018587 | none |
| rs78056098 | none |
| rs949971 | LOC105374037 |
| rs10404377 | LOC105374037 |
| rs482905 | none |
| rs6501525 | LOC124900392 |
| rs66753613 | none |
| rs6797051 | none |
| rs7514702 | PLA2G4A |
| rs12555298 | none |
| rs2585223 | ADAMTS17 |
| rs584122 | none |
| rs61972390 | CLYBL-AS3 |
| rs7148504 | CYP46A1 |
| rs76531781 | DNAH11 |
| rs789068 | LOC107985165 |
| rs9864379 | LOC107985165 |

| IVs of acne vulgaris are matched to genes | |
| --- | --- |
| SNP | gene |
| rs10734852 | BORCS5 |
| rs10941664 | FGF10 |
| rs11022666 | none |
| rs11227289 | none |
| rs11231890 | CDC42BPG |
| rs130291 | TIMP3 |
| rs1481362 | none |
| rs174594 | FADS2 |
| rs185094 | LOC105378977 |
| rs2522051 | IRF1-AS1 |
| rs2600262 | none |
| rs2696958 | PPP1R12B |
| rs3118181 | LAMC2 |
| rs34560261 | SEMA4B |
| rs37776 | none |
| rs4760791 | LOC105369832 |
| rs630904 | none |
| rs6498135 | CIITA |
| rs7410766 | none |
| rs8078102 | CCDC57 |
