# Supplementary figures and images for: Mendelian randomization analysis reveals an independent causal relationship between four gut microbes and acne vulgaris
Source: Front Microbiol. 2024 Feb 2;15:1326339. doi: 10.3389/fmicb.2024.1326339 (PMC10869500; doi:10.3389/fmicb.2024.1326339)

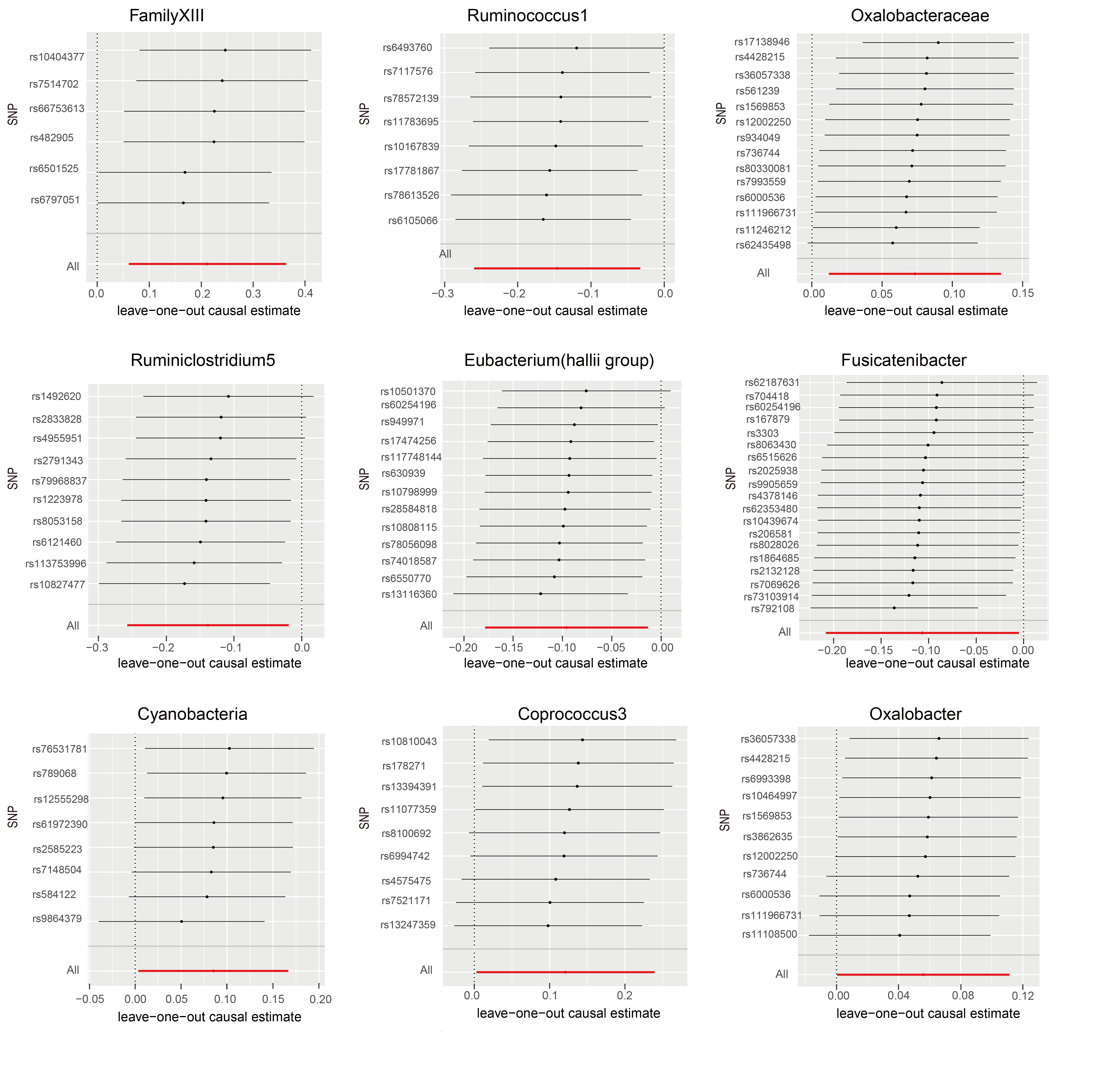

Supplement: Supplementary file 2 [file Image_1.TIFF]
